# The nematode parasite *Steinernema hermaphroditum* is pathogenic to *Drosophila melanogaster* larvae without activating their immune response

**DOI:** 10.17912/micropub.biology.000944

**Published:** 2023-09-25

**Authors:** Tien Huynh, Damien O'Halloran, John Hawdon, Ioannis Eleftherianos

**Affiliations:** 1 Department of Biological Sciences, The George Washington University, Washington, DC, USA; 2 Department of Microbiology, Immunology, and Tropical Medicine, The George Washington University, Washington, DC, USA

## Abstract

Entomopathogenic nematodes are commonly used to control insect pest populations in the field. They also contribute substantially to understanding the molecular basis of nematode pathogenicity and insect anti-nematode immunity. Here, we tested the effect of the entomopathogenic nematode *Steinernema hermaphroditum* on the survival and immune signaling regulation of *Drosophila melanogaster* wild type larvae. Our results indicate that *S. hermaphroditum* infective juveniles are pathogenic toward *D. melanogaster* larvae, but they fail to activate certain immune pathway readout genes. These findings imply that *S. hermaphroditum* employs mechanisms that allow these parasitic nematodes to interfere with the *D. melanogaster* immune system.

**Figure 1.**
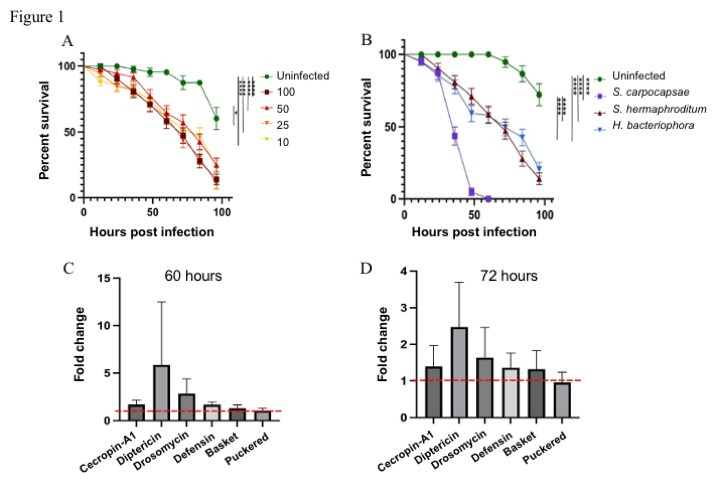
**
The entomopathogenic nematode *Steinernema hermaphroditum* is pathogenic to *Drosophila melanogaster* larvae but fails to activate the immune response:** (A) Percent survival of *D. melanogaster* larvae following infection with four different concentrations of *S. hermaphroditum* (100, 50, 25, and 10 infective juveniles, IJs). Uninfected larvae were treated with sterile water only (****P<0.0001, *P<0.05). (B) Percent survival of *D. melanogaster* larvae following infection with 100 *Steinernema carpocapsae* , *Steinernema hermaphroditum* and *Heterorhabditis bacteriophora* IJs. Uninfected larvae were treated with sterile water only (****P<0.0001). Expression analysis of immune pathway gene readouts in *D. melanogaster* larvae at (C) 60 hours and (D) 72 hours following infection with 100 *S. hermaphroditum* IJs. The horizontal red dotted line indicates the baseline gene expression in uninfected insects.

## Description

The entomopathogenic nematodes (EPNs) *Heterorhabditis* sp. and *Steinernema* sp. form excellent experimental models to dissect the molecular basis of nematode parasitism in relation to the insect immune system (Castillo *et al* ., 2011). The study of the interaction between insect model hosts and EPNs provides insights into the mechanisms underlying nematode virulence and host immunity, and enables whole-animal, high-throughput infection assays (Hallem *et al* ., 2007). Understanding the molecular mechanisms of host responses to EPNs will potentially lead to the development of innovative means for exploiting EPN as biocontrol agents through the implementation of pest management strategies involving the use of EPN molecules with immunomodulatory properties (Brivio and Mastore, 2018) .

The EPN *Steinernema hermaphroditum* has been isolated from insect hosts and produces hermaphrodites in the first generation and males and females in the second generation (Stock *et al* ., 2004). This nematode parasite harbors the mutualistic *Xenorhabdus* *griffiniae* bacteria. The nematodes go through four larval stages (J1-J4) before they mature into adults, which produce eggs containing fertilized embryos. The second larval stage develops into the infective juvenile (IJ) stage, which is developmentally arrested and equivalent to the *C. elegans* dauer stage. The IJs carry the *X. griffiniae* cells in a specialized intestinal vesicle (Bird and Akhurst, 1983) . When the *S. hermaphroditum* IJs infect suitable insect hosts, the nematodes and their bacteria secrete virulence factors and effector molecules that neutralize the insect immune response and destroy vital insect tissues. The nematodes feed on the insect cadaver and when the nutrients are depleted, a new generation of IJs appears. The newly developed IJs eventually exit the insect carcass to locate and invade susceptible hosts (Cao *et al* ., 2022).

The fruit fly *Drosophila melanogaster* is a versatile model for studying the evolution of microbial pathogenicity and host defense (Yu *et al* ., 2022). Previous work in *D. melanogaster* has identified the molecular processes that regulate host-pathogen interactions (Younes *et al* ., 2020). The fly has also been used recently as model to understand the molecular basis of nematode pathogenicity and host anti-nematode innate immunity. The anti-EPN immune response of *D. melanogaster* consists of humoral and cellular immune responses which are linked through the melanization cascade (Ozakman and Eleftherianos, 2021) . Recent studies have further focused on the nature of the EPN excreted-secreted molecules that undermine the fly immune system (Jones *et al* ., 2022; Lima *et al* ., 2022; Parks *et al* ., 2023). In relation to *Steinernema* sp. nematodes, a previous RNA-sequencing analysis in *D. melanogaster* larvae identified several differentially regulated genes encoding factors with cellular immune properties (Yadav *et al* ., 2017). Infection of *D. melanogaster* larvae with *S. carpocapsae* upregulates genes in the two NF-κΒ signaling pathways, Toll and Immune deficiency (Imd), and the phenoloxidase response (Yadav *et al* ., 2018). In addition, *Steinernema feltiae* and *S. carpocapsae* IJs release toxic proteins that compromise adult fly survival through immunosuppression (Lu *et al* ., 2017; Chang *et al* ., 2019).

In the current work, we have explored the pathogenicity of *S. hermaphroditum* IJs toward *D. melanogaster* larvae. We found significant differences between the survival curves of *S. hermaphroditum* infected larvae and control individuals, and nematode infected larvae died significantly faster than controls (Mantel-Cox, *df* = 1, *P* < 0.0001) ( Figure 1A ). However, there were no significant differences between the survival curves of *D. melanogaster* larvae infected with four different concentrations of *S. hermaphroditum* IJs ( Figure 1A ). Specifically, *S. hermaphroditum* IJs caused 50% mortality of *D. melanogaster* larvae between 60 to 72 hours post infection and killed on average approximately 75% of the infected individuals by the end of the monitoring period ( Figure 1 ).

We then examined the pathogenicity between three EPN species. We found that *S. carpocapsae* nematodes were significantly more pathogenic than *S. hermaphroditum* and *H. bacteriophora* (Mantel-Cox, *df* = 1, *P* < 0.0001), while *H. bacteriophora* and *S. hermaphroditum* were equally pathogenic toward *D. melanogaster* larvae (Mantel-Cox, *df* = 1, *P* = 0.3108). Also, *S. hermaphroditum* IJs caused 50% larval mortality at 60 to 72 hours post infection, compared to 36 hours for *S. carpocapsae* nematodes ( Figure 1B ).

To assess whether *S. hermaphroditum* activates the *D. melanogaster* immune response, we examined the transcriptional regulation of immune signaling pathway readout genes at 60 and 72 hours post nematode infection (Figures 1C and 1D). Although there was a 6-fold upregulation of *Diptericin* in *S. hermaphroditum* infected larvae at 60 hours post infection, it was not significantly different compared to uninfected controls (ANOVA, *F* = 1.333, *P* = 0.1488). Similarly, none of the six genes showed significant change in expression between the *S. hermaphroditum* -infected larvae and the uninfected controls for both tested time points.

We find that 10 *S. hermaphroditum* IJs are sufficient to kill *D. melanogaster* wild type larvae within four days of infection. It is worth noting that here we tested the infectivity of the *S. hermaphroditum* - *X. griffiniae* nematode-bacteria complex, therefore we expect that both symbiotic partners contribute to pathogenicity toward *D. melanogaster* . Curiously, infecting larvae with a higher number of nematodes did not substantially affect the time of death. We have previously found a similar pattern for *D. melanogaster* adults infected with symbiotic *Heterorhabditis bacteriophora* nematodes, where all flies succumbed within four days of infection with different numbers of nematodes ranging from 10 to 100 (Castillo *et al* ., 2012). The current results may suggest that exposing the larvae to more IJs does not lead to more IJs attempting to infect or larvae cannot die any faster than they do with a dose of 10. Alternatively, there could be density dependent effects on the nematodes that prevent development of more nematodes at the higher doses. These possibilities will be further explored in future studies.

Also, an interesting observation is that *S. hermaphroditum* IJs are less pathogenic to *D. melanogaster* larvae compared to the closely related *S. carpocapsae* and equal pathogenic to *H. bacteriophora* . These findings indicate that *S. hermaphroditum* / *X. griffiniae* and *S. carpocapsae* / *X. nematophila* may utilize distinct infection strategies during *D. melanogaster* larval infection. Instead, these data imply that the *D. melanogaster* immune response is probably regulated differently by *S. hermaphroditum* and *H. bacteriophora* IJs. Indeed, here we find that infection of *D. melanogaster* larvae with *S. hermaphroditum* IJs fails to induce the expression of readout genes in the two NF-κB pathways, Imd and Toll, and the JNK pathway. However, our previous work has revealed that injection of *D. melanogaster* with *S. carpocapsae* excreted-secreted products upregulates the expression of antimicrobial peptide-encoding genes in larvae and reduces the activity of the phenoloxidase enzyme in their hemolymph (Dziedziech *et al* ., 2020; Jones et al. 2022). Similarly, injection of recombinant *H. bacteriophora* UDP-glycosyltransferase into *D. melanogaster* adults decreases the upregulation of antimicrobial peptide genes associated with both the Toll and Imd pathways (Kenney *et al* ., 2020). The suppressive effect of immune signaling activation observed in larvae infected with *S. hermaphroditum* IJs could also be attributed to their symbiotic *X. griffiniae* bacteria. Previous research has demonstrated that *X. nematophila* infection induces antibacterial peptides in *D. melanogaster* but inhibits phagocytosis and the melanization response (Ozakman and Eleftherianos, 2020) . It remains to be shown whether prevention of immune signaling activation by the *S. hermaphroditum-X. griffiniae* complex impairs host reactions directed against the nematodes and their symbiotic bacteria (Bobardt *et al* ., 2020; Eleftherianos and Heryanto, 2021).

In conclusion, here we explored the interaction between the EPN *S. hermaphroditum* with the model insect host *D. melanogaster* . The obtained results can potentially serve as a foundation for future in-depth studies to elucidate the molecular basis of nematode-bacterial symbiosis, nematode parasitism, and host anti-nematode immunity. Information on *S. hermaphroditum* pathogenicity will further highlight the potential of these nematodes to serve as biological control agents against insect pests and disease vectors. For further studies, the generation of axenic *S. hermaphroditum* (devoid of *X. griffiniae* bacteria) will determine the nematode strategies that promote pathogenesis and interfere with host defenses during infection. Also, identification of the specific *S. hermaphroditum* infection factors using genome-wide transcriptome analyses together with gene silencing or genome editing approaches will define the genetic regulation of EPN infectivity. Considering thatthe *Drosophila* innate immune system is evolutionary conserved, this research will facilitate modeling parasitic nematode processes and anti-nematode immune reactions in vertebrates, including humans (Silverman and Maniatis, 2001; Stock, 2005; Castillo *et al* ., 2011).

## Methods

**Fly stocks.** *Drosophila melanogaster* late second to early third instar larvae from parent strain Oregon-R (Bloomington *Drosophila* Stock Center; 33055) were grown on instant *Drosophila* diet (Formula 4–24 *Drosophila* medium) supplemented with yeast (Carolina Biological Supply), incubated at 25 ^o^ C in a 12:12 hour light:dark photoperiodic cycle.

**Nematode stocks.** *Steinernema hermaphroditum* strain CS34 nematodes were gifted from the lab of Paul Sternberg (California Institute of Technology). *Steinernema carpocapsae* and *Heterorhabditis bacteriophora* TT01 exist in our stocks. All nematodes were cultured by infecting *Galleria mellonella* with nematode IJs and incubated at 25 °C in a 12:12 hour light:dark photoperiodic cycle for 7 days before transferring them on a white trap filled with sterile water. IJs selected for infection were within four weeks of collection date.

**Larval infection with nematodes.** *Drosophila melanogaster* second to early third instar Oregon-R larvae were placed individually into the wells of a 96-well plate, which were previously filled with 100 μL of 1.25% agarose gel. Four different concentrations of *S. hermaphroditum* nematodes (10, 25, 50, and 100 IJs) in 10 μL of sterile water were determined through dilution count. Nematode suspensions were pipetted directly onto 12 larvae, the 96-well plates were sealed with Masterclear real-time PCR film (Eppendorf), and holes were pierced for ventilation. Survival of infected larvae was monitored at 12-hour intervals and up to 96 hours. Survival rate was determined by dividing the number of living larvae at each time point by the total number of larvae tested. Larvae that escaped the plate were not considered in the survival rate analysis. For larval infections involving *S. hermaphroditum* , *S. carpocapsae* or *H. bacteriophora* , each *D. melanogaster* larva was infected with 100 IJs of each entomopathogenic nematode suspended in 10 μL of sterile water. For all survival experiments, control larvae were treated with 10 μL of pure water only. Each survival experiment was repeated three times, each containing at least thirty larvae.

**Gene expression analysis.** *Drosophila melanogaster* second to early third instar Oregon-R larvae were infected with 100 *S. hermaphroditum* nematodes as described in the infection protocol above. Live infected larvae and uninfected controls were removed from the 96-well plates and frozen at -80 ^o^ C at 60 and 72 hours post nematode infection or sterile water treatment (controls). RNA was extracted by homogenizing the larvae in TRIzol reagent following the manufacturer’s protocol (Sigma). Concentrations of RNA samples were measured using a NanoDrop spectrophotometer. For cDNA synthesis, RNA samples were normalized at 1000 ng/μL. Reverse transcription and cDNA synthesis were performed using the Applied Biosystems High-Capacity cDNA Reverse Transcription Kit by following the manufacturer's protocol. The cDNA samples were diluted at 1000 ng per 2 μL of sterile water and used for gene expression analysis. Quantitative RT-PCR (qRT-PCR) assays were conducted with the iQ SYBR Green Supermix (Bio-Rad Laboratories) following the manufacturer’s protocol together with gene-specific primers (see Reagents section) and 3.5 ng cDNA in a CFX96 Real-Time System, C1000 Thermal Cycler (Bio-Rad Laboratories). Quantity of mRNA in each sample was normalized to mRNA of the housekeeping gene *RpL32* and presented as a ratio of the value for infected larvae to that of the uninfected controls. Each qRT-PCR experiment was run in biological and technical triplicates and repeated three times.

**Statistical analysis.** Larval survival rate of nematode infected larvae in relation to uninfected controls and comparisons between the different treatments were analyzed using log-rank (Mantel-Cox) and Gehan-Breslow-Wilcoxon tests in GraphPad Prism 9 software. Comparison of gene expression levels between the experimental and control treatments were performed using one-way analysis of variance (ANOVA) and Dunnet’s multiple comparisons tests in GraphPad Prism 9 software.

## Reagents

Primers for qRT-PCR experiments:

**Table:** 

**Gene**	**Accession Number**	**Forward Primer**	**Reverse Primer**
*RpL32*	CG7939	GATGACCATCCGCCCAGCA	CGGACCGACAGCTGCTTGGC
*Cecropin-A1*	CG1365	TCTTCGTTTTCGTCGCTCTC	CTTGTTGAGCGATTCCCAGT
*Diptericin*	CG12763	GCTGCGCAATCGCTTCTACT	TGGTGGAGTTGGGCTTCATG
*Drosomycin*	CG10810	GACTTGTTCGCCCTCTTCG	CTTGCACACACGACGACAG
*Defensin*	CG1385	CGCATAGAAGCGAGCCACATG	GCAGTAGCCGCCTTTGAACC
*Basket*	CG5680	GACAGCTCAGCACCAACACT	GCTTGGCATGGGTTACATTT
*Puckered*	CG7850	GGCCTACAAGCTGGTGAAAG	AGTTCAGATTGGGCGAGATG

## References

[R1] Bird AF, Akhurst RJ. 1983. The nature of the intestinal vesicle in nematodes of the family steinernematidae. Int J Parasitol 13: 599-606.

[R2] Bobardt SD, Dillman AR, Nair MG (2020). The Two Faces of Nematode Infection: Virulence and Immunomodulatory Molecules From Nematode Parasites of Mammals, Insects and Plants.. Front Microbiol.

[R3] Brivio MF, Mastore M (2018). Nematobacterial Complexes and Insect Hosts: Different Weapons for the Same War.. Insects.

[R4] Cao M, Schwartz HT, Tan CH, Sternberg PW (2022). The entomopathogenic nematode Steinernema hermaphroditum is a self-fertilizing hermaphrodite and a genetically tractable system for the study of parasitic and mutualistic symbiosis.. Genetics.

[R5] Castillo JC, Reynolds SE, Eleftherianos I (2011). Insect immune responses to nematode parasites.. Trends Parasitol.

[R6] Castillo JC, Shokal U, Eleftherianos I (2012). A novel method for infecting Drosophila adult flies with insect pathogenic nematodes.. Virulence.

[R7] Chang DZ, Serra L, Lu D, Mortazavi A, Dillman AR (2019). A core set of venom proteins is released by entomopathogenic nematodes in the genus Steinernema.. PLoS Pathog.

[R8] Dziedziech A, Shivankar S, Theopold U (2020). Drosophila melanogaster Responses against Entomopathogenic Nematodes: Focus on Hemolymph Clots.. Insects.

[R9] Eleftherianos I, Heryanto C (2021). Transcriptomic Insights into the Insect Immune Response to Nematode Infection.. Genes (Basel).

[R10] Hallem EA, Rengarajan M, Ciche TA, Sternberg PW (2007). Nematodes, bacteria, and flies: a tripartite model for nematode parasitism.. Curr Biol.

[R11] Jones K, Tafesh-Edwards G, Kenney E, Toubarro D, Simões N, Eleftherianos I (2022). Excreted secreted products from the parasitic nematode Steinernema carpocapsae manipulate the Drosophila melanogaster immune response.. Sci Rep.

[R12] Kenney E, Yaparla A, Hawdon JM, O'Halloran DM, Grayfer L, Eleftherianos I (2020). A putative UDP-glycosyltransferase from Heterorhabditis bacteriophora suppresses antimicrobial peptide gene expression and factors related to ecdysone signaling.. Sci Rep.

[R13] Lima AK, Dhillon H, Dillman AR (2022). ShK-Domain-Containing Protein from a Parasitic Nematode Modulates Drosophila melanogaster Immunity.. Pathogens.

[R14] Lu D, Macchietto M, Chang D, Barros MM, Baldwin J, Mortazavi A, Dillman AR (2017). Activated entomopathogenic nematode infective juveniles release lethal venom proteins.. PLoS Pathog.

[R15] Ozakman Y, Eleftherianos I (2020). Immune interactions between Drosophila and the pathogen Xenorhabdus.. Microbiol Res.

[R16] Ozakman Y, Eleftherianos I (2021). Nematode infection and antinematode immunity in Drosophila.. Trends Parasitol.

[R17] Parks SC, Okakpu OK, Azizpor P, Nguyen S, Martinez-Beltran S, Claudio I, Anesko K, Bhatia A, Dhillon HS, Dillman AR (2023). Parasitic nematode secreted phospholipase A(2) suppresses cellular and humoral immunity by targeting hemocytes in Drosophila melanogaster.. Front Immunol.

[R18] Silverman N, Maniatis T (2001). NF-kappaB signaling pathways in mammalian and insect innate immunity.. Genes Dev.

[R19] Stock SP (2005). Insect-parasitic nematodes: from lab curiosities to model organisms.. J Invertebr Pathol.

[R20] Stock SP, Griffin CT, Chaerani R. 2004. Morphological and molecular characterisation of Steinernema hermaphroditum n. sp.(Nematoda: Steinernematidae), an entomopathogenic nematode from Indonesia, and its phylogenetic relationships with other members of the genus. Nematology 6: 401-12.

[R21] Yadav S, Daugherty S, Shetty AC, Eleftherianos I (2017). RNAseq Analysis of the Drosophila Response to the Entomopathogenic Nematode Steinernema.. G3 (Bethesda).

[R22] Yadav S, Gupta S, Eleftherianos I (2018). Differential Regulation of Immune Signaling and Survival Response in Drosophila melanogaster Larvae upon Steinernema carpocapsae Nematode Infection.. Insects.

[R23] Younes S, Al-Sulaiti A, Nasser EAA, Najjar H, Kamareddine L (2020). Drosophila as a Model Organism in Host-Pathogen Interaction Studies.. Front Cell Infect Microbiol.

[R24] Yu S, Luo F, Xu Y, Zhang Y, Jin LH (2022). Drosophila Innate Immunity Involves Multiple Signaling Pathways and Coordinated Communication Between Different Tissues.. Front Immunol.

